# Impact of loneliness on health‐related factors in Australia during the COVID‐19 pandemic: A retrospective study

**DOI:** 10.1111/hsc.13948

**Published:** 2022-07-28

**Authors:** Shradha Vasan, Elisabeth Lambert, Nina Eikelis, Michelle H. Lim

**Affiliations:** ^1^ Iverson Health Innovation Research Institute, Swinburne University of Technology Hawthorn Australia; ^2^ Centre for Mental Health Swinburne University of Technology Hawthorn Australia

**Keywords:** COVID‐19, health literacy, loneliness, mental health, physical health, social health

## Abstract

COVID‐19 pandemic and its associated social and physical distancing restrictions may have had a severe impact on health. In the present study, we investigate the changes in physical, social and mental health, as well as the health literacy of Australians subsequent to the onset of COVID‐19 pandemic, and examine the influence of loneliness on these health‐related factors. Using a retrospective cross‐sectional study design, 607 Australian adults completed a self‐report online survey which assessed their health‐related factors before and after onset of the COVID‐19 pandemic (data collected between June 2020 to November 2020). Australians reported statistically significant increase in a number of (poorer) health‐related factors (e.g., weight gain, sleeping difficulties, poor somatic health, higher loneliness, more issues navigating the healthcare system) post onset of COVID‐19 pandemic. Further, after adjusting for covariates, higher loneliness during pandemic predicted poorer health‐related outcomes (e.g., more somatic health complaints, poorer quality of diet, poorer social support for health). The COVID‐19 pandemic and its associated social and physical distancing restrictions may have contributed towards poorer health‐related factors among Australian adults. Further, increased loneliness during the pandemic may have further worsened physical health and health literacy outcomes among Australians.

**What is known about the topic**

- Several social and physical distancing restrictions were implemented in Australia to reduce the spread of coronavirus infection and lessen the burden on healthcare system.
- Loneliness is as a negative by‐product of the COVID‐19 pandemic.
- Prior to the COVID‐19 pandemic, loneliness was recognised as a growing public health problem with robust empirical evidence to demonstrate its the negative impact on health.

**What this paper adds**

- Our findings suggest COVID‐19 pandemic had a severe impact on physical health, social and mental health, and health literacy of Australians.
- Increased loneliness during the pandemic contributed towards poorer physical health and lower health literacy among Australians.
- Loneliness is often considered a precursor to more severe mental health symptoms, as such, our findings could help clinicians and public health officials in better understanding the detrimental impact of loneliness on health‐related factors and developing more targeted interventions and policies to manage the short‐ and long‐term negative impact of loneliness on health during the COVID‐19 pandemic in Australia.

Loneliness is a major consequence of the novel coronavirus (COVID‐19) pandemic (Holmes et al., 2020). To control the spread of infection and its considerable negative health effects, physical distancing restrictions were implemented worldwide (Wilder‐Smith & Freedman, 2020). These restrictions led to changes in one's social routines, including working from home, closures of social and sporting venues, restricted international and interstate travel, among others (Wilder‐Smith & Freedman, 2020). Abovementioned restrictions played an integral part in ‘flattening the curve’ by reducing the spread of infection and lessening the burden on the healthcare system (Shakespeare‐Finch et al., 2020). However, for many Australians the economic and social changes associated with these restrictions have come at considerable personal cost, including the onset and worsening of health‐related issues (Australian Medical Association, 2020; Holmes et al., 2020; Killgore et al., 2020). Indeed, researchers found a strong relationship between onset of the pandemic, associated restrictions, and increase in negative social and mental health symptoms (Australian Medical Association, 2020; Kim & Jung, 2021; Kiuchi et al., 2020; Lim et al., 2022; Nguyen et al., 2020; Rossell et al., 2021; Stanton et al., 2020; Varma et al., 2021; Wang et al., 2020), including loneliness (Bu et al., 2020a, 2020b; Okruszek et al., 2020). Further, findings from Stanton et al. (2020) and Neill et al. (2020) reported poorer physical health symptoms (e.g., lower physical activity, poorer sleep, higher smoking, and alcohol use) among Australians during the COVID‐19 pandemic. In addition to physical health, social and mental health, health literacy is also an independent determinant of health‐related outcomes (Geboers et al., 2016). Health literacy involves the use of cognitive and social skills in order to gain access to, evaluate, and engage with health‐related information to maintain good health, enable disease prevention, and make informed health‐related decisions (World Health Organisation, 1998). Nonetheless, there is a paucity of research surrounding the impact of the pandemic on health literacy in Australia.

Loneliness is a subjective and adverse experience which arises due to differences between an individual's actual and desired quality of social relationships (Peplau & Perlman, 1982). It is highly prevalent. In fact, a 2018 survey found one in four Australians (between the ages of 18 and 89) experienced loneliness (Abbott et al., 2018). As mentioned previously, loneliness has been identified as a negative by‐product of the pandemic (Holmes et al., 2020; Killgore et al., 2020). For some people, experience of loneliness may be temporary, that is, a direct consequence of limited social interaction during the pandemic. However, for others it may be chronic, and changes associated with the pandemic may exacerbate their feelings of loneliness.

Prior to the COVID‐19 pandemic, loneliness was recognised as a growing public health problem (Cacioppo & Cacioppo, 2018a; Lim et al., 2020). There is robust cross‐sectional and longitudinal evidence to demonstrate the negative impact of loneliness on health outcomes (for a comprehensive review; see Lim et al., 2020). Likewise, there is substantial research outlining the potential underlying mechanisms through which loneliness may affect health (for theories explaining how loneliness may perpetuate and affect physical health and other health‐related factors; see Cacioppo & Cacioppo, 2018b; Cacioppo & Hawkley, 2009). For brevity, in this paper we will be focusing on only three different aspects of health‐related outcomes or health‐related factors, including (1) physical health, (2) social health (defined as an individual's ability to interact, develop meaningful relationships, and act as a functioning member of their community; Renne, 1974) and mental health, and (3) health literacy. Before the onset of the COVID‐19 pandemic, researchers found a consistent relationship between higher loneliness and poor physical health indicators (e.g., increased cardiovascular issues, reduced physical activity, sleeping difficulties; Cacioppo, Hawkley, Berntson, et al., 2002; Cacioppo, Hawkley, Crawford, et al., 2002; Hawkley et al., 2006; Hawkley et al., 2009; Hawkley et al., 2010; Kurina et al., 2011). A number of these physical health indicators are associated with cardiovascular and metabolic disease development (Mozaffarian et al., 2008). Further, loneliness covaries with a variety of social and mental health indicators, including, social isolation (de Jong Gierveld & Havens, 2004; de Jong Gierveld et al., 2006; Matthews et al., 2016 ‐ social health indicator), social anxiety and depression (Cacioppo et al., 2010; Lim et al., 2016 ‐ mental health indicators). While limited, researchers have reported an association between increased loneliness and poor health literacy among older adults (Bennett et al., 2012; Geboers et al., 2016). It is important to consider health literacy when investigating the impact of loneliness on health and health‐related factors as extensive literature documents the association between lower health literacy and poorer health outcomes (Geboers et al., 2016; Marvanova et al., 2011; Miller, 2016; Mitchell et al., 2012). To the best of our knowledge, few studies have investigated the changes in different health‐related factors during the COVID‐19 pandemic, and no published studies have specifically examined how changes in loneliness (i.e., before and during the pandemic) may be associated with changes in the physical health and health literacy (before and during the pandemic) of Australians, after accounting for co‐occurring social and mental health symptoms for loneliness.

Therefore, the primary aims of this study were to:

1. Investigate the changes in physical, social and mental health, as well as health literacy, of Australians before and during the COVID‐19 pandemic.
2. Examine how changes in loneliness were associated with changes in physical health and health literacy, of Australians, after controlling for the influence of concomitant social and mental health indicators (social isolation, social anxiety, depressive symptomology).

## 1 METHOD

Swinburne University Human Research Ethics Committee granted ethical approval (approval number 20202950–4247) for the current study. Using a retrospective cross‐sectional study design, 607 Australian residents between the ages of 18 to 63 completed an online survey hosted on Qualtrics (survey platform). Survey was distributed through word of mouth, advertisements on social networks, online forums and via Swinburne University research training programme for undergraduate first year students. The sample consisted of community members and undergraduate students at Swinburne University. First year undergraduate students were compensated with course credit in return for participation and community members went into a draw to win two $50 gift cards. An explanatory statement was provided, and informed consent was obtained prior to participants completing the survey. Data were collected between June 2020 and November 2020; the time‐period during which the pandemic related restrictions was in place in Australia.

As we were investigating *changes* (before and during the pandemic) in health‐related outcomes of Australians, we asked the participants to *retrospectively* self‐report their health‐related factors before onset of the COVID‐19 pandemic. Throughout the online survey we consistently defined ‘before onset of the COVID‐19 pandemic’ as the time‐period prior to 11th of March 2020 (i.e., date on which World Health Organisation declared coronavirus a pandemic; World Health Organisation, 2020a). To capture participants' health‐related factors during the pandemic, we asked the respondents to report their ‘current’ health‐related factors. Throughout present paper, the term ‘before onset of the COVID‐19 pandemic’ is also referred to and used interchangeably with terms such as, ‘pre’ or ‘prior to onset of the COVID‐19 pandemic’. Likewise, terms ‘current’ or during the pandemic’ will be referred to and used synonymously with terms, such as, ‘after onset of the COVID‐19 pandemic’, ‘post onset of the COVID‐19 pandemic’ in this article. To maintain consistency and accuracy across participants, we used the same questionnaires to record responses for health‐related outcomes for ‘before’ and ‘after onset of the COVID‐19 pandemic’. The online survey completed by participants consisted of the following scales measuring an array of health‐related factors.

### 1.1 Materials

Demographic information including age, gender, ethnicity, marital status, living status, education level, use of English as a primary language, and sexual orientation were obtained. To assess the personal impact of the pandemic, participants were asked to report if they had contracted/were affected by coronavirus at the time of the survey. Using single‐item questions, participants self‐reported for physical health indicators such as weight and height. Scores for physical activity and alcohol use were calculated using participant's responses for frequency and intensity of use. Body mass index (BMI) values were calculated from the self‐reported weight and height measurements. Additional information on how these health‐related outcomes were measured is provided in Supplementary Material.

#### 1.1.1 Other physical health measures

##### 1.1.1.1 Insomnia Severity Index (ISI)

The ISI is a 7‐item self‐report measure which assesses the severity and effect of insomnia or poor sleep over a 2‐week period (e.g., *‘Difficulty falling asleep’*) (Morin et al., 2011). Scores are rated on a 5‐point Likert‐type scale, ranging from 0 (*no problem*) to 4 (*very severe problem*). Total scores ranged from 0 to 28. Higher scores indicated increased sleeping difficulties. ISI is shown to have very strong internal reliability with Cronbach's α values ranging from 0.90 to 0.91 (Morin et al., 2011).

##### 1.1.1.2 Physical Health Questionnaire (PHQ)

The PHQ is developed to be a brief self‐report scale of somatic health symptoms (Schat et al., 2005). PHQ consists of 14‐items and assess four aspects of physical health symptoms: gastrointestinal problems, headaches, sleep disturbance and respiratory infections. Items are rated on a 7‐point frequency scale, an example includes ‘*How often have you suffered from an upset stomach (indigestion)?’*. A total somatic health score is calculated, scores ranged from 7 to 98 with higher scores indicating more somatic health issues. The scale has good psychometric properties (Schat et al., 2005).

##### 1.1.1.3 Diet Quality Tool (DQT)

The DQT is a 13‐item scale which assess whether the participant's dietary habits or quality of diet is in line with the recommended nutritional guidelines for prevention of cardiovascular disease (e.g., vegetable and fruit intake, saturated or total fat intake). Total scores are calculated by summing the responses for each of the question, scores range from 0 to 130, with the higher scores indicating a higher level of compliance with the proposed dietary guidelines for prevention of cardiovascular disease and consequently higher diet quality (O'Reilly & McCann, 2012).

##### 1.1.1.4 Perceived General Health

Perceived general health was measured using a single question ‘*In general would you say that your health is excellent, very good, good, fair or poor*?’ on a scale 1 to 5 (Schat et al., 2005). Higher scores on this question indicate better perceived general health.

#### 1.1.2 Social and mental health measures

##### 1.1.2.1 UCLA Loneliness Scale (UCLA‐LS)

The UCLA‐LS is a 20‐item measure using a 1 (*Never*) to 4 (*Always*) Likert‐type scale. This measure assesses the levels of loneliness over a 1‐month period (Russell, 1996). It consists of nine positively worded items (e.g., ‘*How often do you feel that you are “in tune” with the people around you?’* ) and eleven negatively phrased questions (e.g., ‘*How often do you feel that people are around you but not with you?’*) (Russell, 1996). Total scores range from 20 to 80. Higher scores indicate higher levels of loneliness. The UCLA‐LS has demonstrated excellent reliability and convergent validity with related constructs (Russell, 1996).

##### 1.1.2.2 Lubben Social Network Scale (LSNS)

The LSNS is a self‐reported short form that assesses the frequency and quality of social engagement. The scale employs a 0 (*none*) to 5 (*nine or more*) Likert‐type scale and includes 6‐items (e.g., *How many relatives do you see or hear from at least once a month?*) (Lubben, 1988). Higher scores indicate larger social networks and lower risk of social isolation (total score range 0 to 30). The 6‐item version of the scale has demonstrated adequate levels of reliability (Lubben, 1988).

##### 1.1.2.3 Social Interaction Anxiety Scale (SIAS)

We used the straightforward version of the SIAS, which includes 17‐items and employs a 0 (*Not at all characteristic of me*) to 4 (*Extremely characteristic of me*) Likert‐type scale (Brown et al., 1997). This is a modified version of the 20‐item SIAS, where only the straightforward (negatively worded) items are used. Rodebaugh et al. (2007) recommend using only the straightforward 17‐items as they are the better indicators of social interaction anxiety, whereas the three positively worded or reverse scored items tap into the construct of extraversion more closely. The measure involves self‐statements describing one's thoughts, feelings and factors in social situations. An example item includes ‘*I become tense if I have to talk about myself or my feelings’*. Total scores can range from 0 to 68. Higher scores indicate higher levels of social interaction anxiety. The SIAS has demonstrated excellent reliability and construct validity (Brown et al., 1997).

##### 1.1.2.4 Centre for Epidemiological Studies – Depression (CES‐D)

The CES‐D is a 20‐item measure of depression symptoms and employs a 0 (*rare or none of the time*) to 3 (*Most or all of the time*) Likert‐type scale (Radloff, 1977). The measure involves answering questions about the ways in which a person may have behaved or felt in the last 7 days (e.g., *I did not feel like eating; my appetite was poor*). Total scores range from 0 to 60, with higher score indicating increased experience of depressive symptomology. The CES‐D has strong internal reliability (Radloff, 1977). For CES‐D, we only collected post onset of the COVID‐19 pandemic data.

#### 1.1.3 Health literacy scale

##### 1.1.3.1 Health Literacy Questionnaire (HLQ)

The HLQ consists of 44 questions, where health literacy is defined as the use of cognitive and social skills in order to gain access to, evaluate, and engage with health‐related information in order to promote and maintain good health (Osborne et al., 2013). It consists of nine independent and conceptually distinct scales or domains, which together provides insight into the respondents understanding of, engagement with, and use of health‐related services (Osborne et al., 2013). The nine scales include: (1) Feeling understood and supported by healthcare providers; (2) Having sufficient information to manage my health; (3) Actively managing my health; (4) Social support for health; (5) Appraisal of health information; (6) Ability to actively engage with healthcare providers; (7) Navigating the healthcare system; (8) Ability to find good health information; and (9) Understanding health information well enough to know what to do.

There are four to six items in each scale. The first five scales employ a 1 (*strongly disagree*) to 4 (*strongly agree*) Likert type measurement. Scales six to nine use a 1–5 anchor type (*cannot do, very difficult, quite difficult, quite easy, very easy*). Nine individual scores are acquired by averaging the items within each of the scales, where higher scores indicate better understanding and use of health‐related information (i.e., higher health literacy) for that particular domain. No total scores are calculated for HLQ as that could possibly mask specific needs in particular health literacy domains (Osborne et al., 2013). The questionnaire has shown excellent psychometric properties and has been validated for use in a variety of different settings and populations (e.g., clinical, home, community care, adults 18 to 64 years and over; Elsworth et al., 2016; Kolarcik et al., 2017; Osborne et al., 2013; Richtering et al., 2017).

### 1.2 Data analytic approach

The data were analysed using IBM Statistical Package for Social Sciences Version 27.0. All the data cleaning and assumption testing information for the statistical techniques used in this study are provided in Supplementary Material. The first research question – investigate the changes in physical health, social and mental health, as well as health literacy, of Australians before and during the COVID‐19 pandemic – was answered using paired samples *t*‐tests. The second research question – associations between changes in loneliness and changes in health‐related factors of Australians subsequent to the onset of COVID‐19 pandemic – was answered using Hierarchical Multiple Regression. The predictor variable was change (score) in loneliness. There were multiple outcome or dependent variables, including change (scores) in physical health indicators (i.e., weight, BMI, somatic health, perceived general health, sleep, diet, alcohol use, physical activity) and the nine health literacy scales. Further, guided by previous empirical research, several covariates were included – age, gender, change (scores) in social anxiety, social isolation (both covary with loneliness; de Jong Gierveld & Havens, 2004; Lim et al., 2020; Lim et al., 2016), post onset of COVID‐19 scores for depressive symptomology (loneliness is often a precursor for depression; Cacioppo et al., 2010; Lim et al., 2020), and *pre* COVID‐19 pandemic score for the outcome variable (to control for any interrelationship with between pre and post onset of the COVID‐19 pandemic scores; Cronbach & Furby, 1970). As specified in Allison (1990) and Dalecki and Willits (1991) ‘change scores’ for predictor, outcome variables, and covariates were calculated by subtracting ‘post onset of the COVID‐19 pandemic’ score value from ‘pre onset of the COVID‐19 pandemic’ score value. A *positive* ‘change score’ indicated *higher* post onset of the COVID‐19 pandemic scores for that variable.

## 2 RESULTS

A total of 607 participants were included in this study. A complete breakdown of participant characteristics is included in Table 1. For categorical variables, percentages are included, and for continuous variables, mean and standard deviation are presented.

**TABLE 1:** Sample characteristics

	Percentage/mean (SD)
**Age (years)**	29.3 (9.2)
**Gender**	
Female	77.4
Male	21.8
Other	0.8
**English as a first language**	
Yes	87.6
No	12.4
**Marital status**	
Single/Never married	41.2
Domestic/defacto relationship	29.9
Married	23.1
Separated/divorced/other	5.8
**Ethnicity**	
White (includes Caucasian, European Australian)	74.2
Asian Australian or Asian (includes Indian, Indian Australian)	12.2
Aboriginal or Torres Strait Islander	0.7
African Australian	1.2
Hispanic or Latino	1.7
Pacific Islander	0.9
Multiple board categories/not specified	9.1
**Sexual orientation**	
Heterosexual	81.8
Homosexual (gay/lesbian)	3.6
Bisexual	9.9
Pansexual/Asexual/not specified	4.6
**Highest level of Education**	
Undergraduate or Bachelor's degree, diploma, graduate certificate	40.8
Postgraduate or Master's degree	13.7
TAFE or trade school	15.1
Secondary school	30.0
Primary school	0.4
**Living status**	
Living with family/housemates/university/relatives	90.3
Living alone	9.7
**Have or had been diagnosed with COVID‐19 at the time of the survey**	
No	100
Yes	
**Participants from Australian States and Territories**	
Victoria	68.6
New South Wales	12.0
Western Australia	4.1
Northern Territory	0.6
South Australia	2.1
Queensland	10.5
Australian Capital Territory	1.3
Tasmania	0.8

*Note*: All the percentages are based on the original data collected – (i.e., before the missing value and multiple imputation analysis was performed).

The descriptive statistics and internal reliabilities (Cronbach's *α*) for all the physical health, social and mental health, and health literacy scales are presented in Table 2.

**TABLE 2:** Reliability testing (Cronbach's *α*) and paired samples *t*‐test for changes in health‐related outcomes before and during COVID‐19 (*N* = 607)

	Mean (*M*)		*SD*		Cronbach's *α*		*M change score*	*SD*	*t*	*p*	Cohen's *d*
	Before COVID‐19	During COVID‐19	Before COVID‐19	During COVID‐19	Before COVID‐19	During COVID‐19					
**Physical health outcomes**											
Weight (Kilograms)	70.69	71.92	15.21	15.99	**—**	**—**	1.23	4.03	−7.52	<0.001***	0.08
Body mass index (Kilogram/metre^2^)	24.81	25.23	4.75	4.99	**—**	**—**	0.42	1.41	−7.41	<0.001***	0.09
Alcohol use	5.58	5.82	6.51	6.56	**—**	**—**	0.25	5.42	−1.12	0.26	0.04
Physical activity	7.92	7.26	4.75	3.37	**—**	**—**	−0.64	4.01	3.95	<0.001***	0.16
Perceived general health	3.51	2.98	0.87	0.90	**—**	**—**	−0.53	0.81	16.0	<0.001***	0.60
Somatic health (PHQ)	41.01	43.27	10.6	12.02	0.83	0.86	2.25	8.56	−1.57	<0.001***	0.20
Sleep (ISI)	8.56	10.88	5.25	5.33	0.87	0.88	2.32	4.81	−2.70	<0.001***	0.44
Diet (DQT)	67.40	67.02	15.95	15.41	**—**	**—**	−0.38	8.18	1.14	0.26	0.02
**Social and mental health outcomes**											
Loneliness (UCLA‐LS)	42.15	46.02	9.76	10.0	0.93	0.94	3.87	7.61	−12.53	<0.001***	0.40
Social anxiety (SIAS)	23.08	24.71	12.71	13.09	0.94	0.95	1.63	6.70	−5.98	<0.001***	0.13
Social isolation (LSNS)	16.46	15.24	5.02	4.95	0.81	0.84	−1.23	2.74	11.02	<0.001***	0.25
**Health literacy scales**											
Feeling understood and supported by healthcare providers	3.01	3.04	0.56	0.60	0.91	0.94	0.029	0.36	−1.99	0.05*	0.05
Having sufficient information to manage my health	3.09	3.06	0.42	0.48	0.91	0.94	−0.03	0.36	2.23	0.03*	0.07
Actively managing my health	2.92	2.94	0.48	0.54	0.91	0.94	0.02	0.40	−1.36	0.18	0.04
Social support for health	3.01	3.03	0.50	0.57	0.91	0.94	0.02	0.32	−1.7	0.08	0.04
Appraisal of health information	2.57	2.97	0.45	0.49	0.91	0.94	0.11	0.38	−6.96	<0.001***	0.85
Ability to actively engage with healthcare providers	3.86	3.79	0.58	0.68	0.96	0.96	−0.07	0.37	4.28	<0.001***	0.11
Navigating the healthcare system	3.83	3.74	0.55	0.63	0.96	0.96	−0.09	0.37	6.07	<0.001***	0.15
Ability to find good health information	3.96	3.92	0.52	0.57	0.96	0.96	−0.04	0.32	3.38	0.001**	0.07
Understand health information well enough to know what to do	4.05	4.04	0.53	0.58	0.96	0.96	−0.02	0.30	1.28	0.20	0.02

*Note*: Descriptive statistics, reliability testing (Cronbach's α ) and paired samples *t* tests results. Scales without Cronbach's *α* were either single‐item measures (i.e., Weight, BMI) or had a different scoring/anchor points for items in the questionnaire (i.e., Diet, physical activity, alcohol use). As the Health literacy questionnaire has two scale points (1 to 4 and 1 to 5), reliability statistics for both the scale points were calculated separately. Significant *p‐*values are bolded, *p < 0.05, **p < 0.01, ***p < 0.001 (two‐tailed tests).

Paired samples *t*‐test were used to investigate changes in health‐related outcomes of Australians prior to and after onset of the COVID‐19 pandemic (all results are presented in Table 2). Participants reported a statistically significant increase in a number of (poorer) physical health indicators, including weight gain, increase in BMI, more somatic health symptoms, and a decrease in physical activity during the pandemic, compared with before the pandemic. These all had small effect sizes (Cohen's *d* values shown in Table 2). Further, respondents reported more sleep issues and poorer perceived general health post onset of the pandemic (medium effect sizes). Likewise, Australian adults reported higher social and mental health issues (higher loneliness, social anxiety, social isolation) during the pandemic, compared with before onset of the pandemic. These had small to medium effect sizes. For health literacy, compared with before the pandemic, respondents reported feeling significantly more understood and supported by healthcare providers (small effect size) and better at appraising and evaluating health information (large effect size) after the onset of the pandemic. Contrastingly, participants reported that they did not feel like they had sufficient information to manage their health, were having issues with actively engaging with healthcare providers navigating the healthcare system, and in finding good health information during the pandemic, in comparison with before the pandemic. These all had small effect sizes.

To answer the second research question, investigating how changes in loneliness are associated with changes in different physical health and health literacy outcomes, we ran individual Hierarchical Multiple Regression models, results are presented in Table 3. For all models, in the first step (in Table 3 presented as Model 1) – age, gender, pre COVID‐19 pandemic scores for the dependent variable were entered. In step two (in Table 3 as Model 2), post onset of the pandemic depressive symptomology and change in social anxiety scores were included. In step three (in Table 3 as Model 3), changes in social isolation scores were entered. In the final step (in Table 3 as Model 4), to ascertain the unique contribution of loneliness during the COVID‐19 pandemic, changes in loneliness scores were entered.

**TABLE 3:** Hierarchical regression analyses for changes in loneliness predicting changes in physical health and health literacy related outcomes (*N* = 607)

Outcome variables	Model 1		Model 2		Model 3		Model 4 (predictor ‐ change in loneliness)	
	*β*	*p*	*β*	*p*	*β*	*p*	*β* [95% confidence interval]	*p*
**Physical health outcomes**								
Weight (Kilograms)	0.06	0.18	0.11	0.01	0.02	0.58	0.02 [−0.03, 0.06]	0.75
Body mass index (Kilogram/metre ^2^)	0.01	0.91	0.10	0.02	0.03	0.52	0.01 [0.00, 0.03]	0.78
Somatic health (PHQ)	−0.26	<0.001	0.22	<0.001	<0.001	0.99	0.11 [0.01, 0.20]	0.01*
Perceived general health	−0.42	<0.001	−0.08	0.030	0.007	0.83	−0.08 [−0.09, −0.07]	0.04*
Sleep (ISI)	−0.40	<0.001	0.14	<0.001	−0.06	0.06	0.11[0.06, 0.16]	0.006**
Diet (DQT)	−0.32	<0.001	−0.09	0.03	0.13	0.001	−0.11 [−0.20, −0.01))	0.02*
Alcohol use	−0.41	<0.001	0.04	0.31	0.09	0.02	−0.04 [−0.10, 0.02]	0.40
Physical activity	−0.71	<0.001	0.02	0.44	0.03	0.30	0.04 [0.00, 0.07]	0.30
**Health literacy scales**								
Feeling understood and supported by healthcare providers	−0.28	<0.001	0.002	0.97	0.02	0.69	0.06 [0.06, 0.06]	0.19
Having sufficient information to manage my health	−0.25	<0.001	−0.006	0.89	−0.02	0.65	−0.03 [−0.04, −0.03]	0.49
Actively managing my health	−0.25	<0.001	−0.029	0.47	0.03	0.41	−0.06 [−0.06, −0.05]	0.22
Social support for health	−0.10	0.01	−0.02	0.69	0.11	0.01	−0.18 [−0.18, −0.17]	<0.001***
Appraisal of health information	−0.32	<0.001	0.01	0.81	−0.04	0.28	0.07 [0.07, 0.07]	0.12
Ability to actively engage with healthcare providers	−0.06	0.15	−0.11	0.009	0.19	<0.001	−0.002 [−0.01, 0.00]	0.96
Navigating the healthcare system	−0.94	0.02	−0.009	0.82	0.15	<0.001	−0.04 [−0.04, −0.04]	0.41
Ability to find good health information	−0.15	<0.001	−0.07	0.09	0.06	0.19	0.10 [0.09, 0.10]	0.04*
Understand health information well enough to know what to do	−0.09	0.04	−0.01	0.77	0.06	0.16	0.10 [0.10, 0.11]	0.03*

*Note*: Hierarchical Multiple Regression analyses results. β = Standardised regression parameter estimates. β values specified in the table are for the last predictor/control variable entered in each model (italicised here). Predictors entered: Model 1 – Age, gender, *pre COVID‐19 scores for the dependent/outcome variable*; Model 2 – Age, gender, pre COVID‐19 scores for the dependent variable, post onset of pandemic depressive symptomology, *change in social anxiety*; Model 3 ‐ Age, gender, pre COVID‐19 scores for the outcome variable, post onset of pandemic depressive symptomology, change in social anxiety, *change in social isolation*; Model 4 ‐ Age, gender, pre COVID‐19 scores for the dependent variable, post onset of pandemic depressive symptomology, change in social anxiety, change in social isolation, *change in loneliness*. Significant *p‐*values are bolded only for unique contribution of loneliness (Model 4); *p < 0.05, **p < 0.01, ***p < 0.001 (two‐tailed tests).

After controlling for covariates, higher experience of loneliness after onset of the COVID‐19 pandemic (positive change score for loneliness) was associated with several poorer physical health outcomes. For example, increase in somatic health complaints (variance explained – 0.8%), increase in sleeping difficulties (variance accounted for 0.8%), poorer quality of diet (variance explained – 0.8%) and overall poorer perceived general health (variance accounted for – 0.5). Further, for health literacy outcomes, we found that increase in loneliness during the pandemic was associated with poorer social support for health (variance explained – 2.2%) post onset of the pandemic. Conversely, positive change score for loneliness during the pandemic were associated with improved ability to find good health care information (variance explained – 0.7%), and increased understanding of health information (variance explained – 0.8%), after onset of the COVID‐19 pandemic.

## 3 DISCUSSION

The aim of this study was to investigate changes in physical health, social and mental health, and health literacy of Australians subsequent to the onset of the COVID‐19 pandemic, and examine the influence of loneliness on these health‐related outcomes.

As anticipated, there were several changes in health‐related factors of Australians following onset of the COVID‐19 pandemic and implementation of associated physical and social restrictions. Consistent with Stanton et al. (2020), we found that Australians were less physically active and experienced more sleep issues during the pandemic. Moreover, post onset of the pandemic, participants reported overall poorer perceived general health, increase in weight and BMI, and experience of more somatic health issues. In other words, they had more respiratory (e.g., cold, flu, bronchitis symptoms) and gastrointestinal track (e.g., indigestion, nausea, diarrhoea) issues and reported more headaches during the COVID‐19 pandemic, compared with the pre pandemic time‐period. There were no significant changes in diet quality among Australians before and during the COVID‐19 pandemic. Unlike Stanton et al. (2020) and Neill et al. (2020), we did not find any changes in alcohol use among Australians prior to and after the onset of the pandemic. Congruent with previous literature (Bu et al., 2020a; Kim & Jung, 2021; Stanton et al., 2020), we found that following onset of the COVID‐19 pandemic, Australians reported a statistically significant increase in a number of social and mental health indicators, including higher experience of loneliness, social isolation and social anxiety. Indeed, it is plausible that the pandemic‐related restrictions were responsible for these changes in health‐related outcomes in Australia. People were lonelier and more socially isolated during the pandemic as they had limited social interactions with other people (e.g., due to restrictions on social gatherings and working from home; Department of Health and Aged Care, 2020). Similarly, closure of gyms and other sporting venues (Department of Health and Aged Care, 2020) may also have contributed towards the reduced physical activity, and weight gain, as well as affected the somatic and general health of the participants.

The findings for changes in health literacy among Australian residents' post pandemic were mixed. For example, after onset of the pandemic, participants felt more understood and supported by their healthcare providers, that is, they felt like they had a trustful and well‐established relationship with at least one healthcare provider, compared with before onset of the pandemic. Further, while respondents had more difficulty finding good healthcare information, for the information they did find or have access to, they were far better at evaluating and critically thinking about this information after onset of the pandemic, compared with before the pandemic. Contrastingly, participants did not feel like they had sufficient information to manage their health, were having issues actively engaging with healthcare providers and had difficulty navigating the healthcare system. The pandemic placed an unprecedented burden on our healthcare system (Quigley et al., 2021), which likely contributed to the difficulty Australians were having engaging and navigating the healthcare system. Equally, the rampant spread of misinformation (Park et al., 2020; World Health Organisation, 2020b) likely added to the issues Australians were having finding good health information. Overall, our results indicated that the COVID‐19 pandemic and its subsequent lifestyle changes may have had a severe detrimental impact on the physical health, social and mental health, and health literacy of Australians.

We also found that changes in loneliness were related to changes in physical health and health literacy outcomes during the COVID‐19 pandemic. After controlling for covariates, higher loneliness during the pandemic was associated with more somatic health complaints, increased sleeping difficulties, poorer quality of diet and overall poorer perceived general health. Further, we found that an increase in loneliness post onset of the COVID‐19 pandemic was significantly related to several health literacy scales. For example, higher loneliness was associated with poorer social support for health. As lonely individuals often feel a deficit in the quality of their social relationships (Cacioppo & Hawkley, 2009; Peplau & Perlman, 1982), it is possible that those who were experiencing increased loneliness during the pandemic were more likely to feel less connected to people who could provide health support, that is, provide social support for their health concerns. More interestingly, we also found that while there was no change in how lonely individuals appraised and evaluated health information, they did report an improved ability to find health care information (e.g., find health information about health problems) and increased understanding of health information (e.g., ability to read and understand health information) during the COVID‐19 pandemic.

Taken together, these findings have a few implications. It is possible that transient feelings of loneliness due to the restrictions during the pandemic and the perceived lack of social support for health concerns encouraged the lonely individual to seek out more information regarding their health management post COVID‐19 pandemic (Cacioppo & Hawkley, 2009). Similarly, it is possible that due to the ‘infodemic’ during the pandemic (Park et al., 2020; World Health Organisation, 2020b), health‐related information was more readily available from a number of different (legitimate and illegitimate) sources. The crucial difference between the changes seen in the ‘general population’ and those who were lonely during the COVID‐19 pandemic was in the way they were appraising and evaluating health‐related information. While in general, participants showed an improvement at identifying and appraising good and reliable sources of health information during the pandemic (as per results of research question one), those who were lonelier during the pandemic did not show any such positive change. This could be quite detrimental for their health, especially considering the rampant spread of misinformation from illegitimate sources (World Health Organisation, 2020b).

### 3.1 Limitations

Nonetheless, some considerations should be made when interpreting the results. Our ‘before onset of the pandemic’ data were collected retrospectively. We acknowledge that retrospective type of data collection may be limited in capturing ‘true’ pre COVID‐19 pandemic health‐related factors and scores. There may have been some retrospective recall bias (Schwarz & Sudman, 2012) in the participant's pre COVID‐19 pandemic responses, as we were relying on our respondents to accurately recall their feelings and factors over long period of time. We did attempt to mitigate this bias to some extent by clearly defining, highlighting and emphasising the ‘pre COVID‐19 pandemic’ time‐period as before 11th of March 2020 (World Health Organisation., 2020a) in every question of the online survey. However, it is likely some retrospective recall bias may be present in the current study.

Additionally, use of the ‘change score’ method has been criticised in the past by some researchers (Cronbach & Furby, 1970). Cronbach and Furby's (1970) concerns included the unreliability of the measurement and the interrelationship between the predictor and the ‘pre‐test’ scores (in present study these include ‘before the COVID‐19 pandemic’ scores). The interrelationship between the predictor and ‘pre‐test’ scores refers to ensuring that the relationship between predictor, and outcome or dependent variable was due to ‘true’ changes or relationships observed between the predictor (i.e., *change*s in loneliness) and outcome variable (i.e., *change* in physical health or health literacy outcome variable); and not due to the association or interrelationship between predictor and ‘pre‐test’ scores (i.e.*, before* the COVID‐19 pandemic score for the physical health or health literacy outcome variable). As recommended by Allison (1990) and Dalecki and Willits (1991), we addressed these issues by calculating the change score using their recommended method and by including before the COVID‐19 pandemic scores for the physical health and health literacy outcome variables as covariates in all the hierarchical regression models (see Table 3).

Lastly, large proportion of our sample was first year undergraduate university students, primarily younger females who spoke English as a first language, highly educated, Caucasian, not living alone and identified as heterosexuals. While undergraduate students are often used in psychosocial research as they are an effective user‐friendly convenience population with lower response bias, administrative cost and associated ease of recruitment (Arnett, 2016; Lucas, 2003), it is important to acknowledge use of student population (where a large proportion are females) may be unrepresentative of the general Australian adult population and result in a more homogenous sample, and thus affecting the generalisability, and by extension the external validity (McTavish & Loether, 2002) of our findings. While this type of data collection provided us with a unique insight into the changes in health‐related outcomes as experienced by Australians during the pandemic and the influence of loneliness on it, we were limited our ability to draw causal inferences and make generalisations, especially since the majority of our sample consisted of younger female adults. Longitudinal data with a more age diverse sample could help provide a deeper insight into how the health‐related outcomes of Australian adults were affected during the COVID‐19 pandemic, and the influence of loneliness on them.

## 4 CONCLUSION

The COVID‐19 pandemic and its associated social and physical distancing restrictions may have come at considerable personal cost for many Australians, including the onset and worsening of several health‐related outcomes. Our findings demonstrate that subsequent to the onset of the pandemic, Australians reported experiencing poorer physical, social and mental health, as well as lower health literacy. Further, we also found that higher experience of loneliness after the onset of the pandemic to be associated with increase in several (poorer) physical health and health literacy outcomes, after adjusting for known covariates. Nonetheless, the study was not without methodological limitations, namely, ‘before onset of the COVID‐19 pandemic’ data were collected retrospectively, which needs to be considered when interpreting the results. Although statistically, these associations had small effect sizes, overall, our results provided several significant insights, especially considering none of the participants reported being directly affected by COVID‐19 (neither contracting the virus nor being required to self‐isolate due to suspected exposure), and overall Australia was moderately impacted (in number of cases and deaths) by the pandemic compared with most other developed countries (World Health Organisation, 2020c).

Findings from our study underscore the significant influence of loneliness on health‐related factors and health literacy among Australian adults during the pandemic. Further, our results demonstrate the importance of analysing and including different health‐related factors and health literacy factors when devising intervention strategies to improve loneliness among Australian adults, especially during the pandemic. Likewise, results from the present study could assist policy makers and public health officials in managing and making more informed decisions about the immediate and long‐term physical health, social and mental health, as well as health literacy needs of Australians following the pandemic. Further, loneliness is often considered a precursor to more severe mental health issues (i.e., depression) and has been associated with earlier mortality and higher morbidity (Holt‐Lunstad et al., 2015). Thus, developing more targeted interventions or policies focusing on modifiable risk factor, such as loneliness, could help clinicians and public health officials in managing the short‐ and long‐term impact of the COVID‐19 pandemic.

## AUTHOR CONTRIBUTIONS

E.L., N.E., M.H.L., S.V. contributed to conceptualisation. E.L., N.E., M.H.L., S.V. contributed to methodology. S.V. contributed to data collection and analysis. S.V.contributed to original draft manuscript preparation. E.L., N.E., M.H.L., S.V. contributed to final manuscript review and editing. E.L., N.E., M.H.L. did supervision. All authors have read and approved the final version of the manuscript.

## CONFLICT OF INTEREST

The authors declare no conflict of interests.

## Supporting information


Appendix S1

## Data Availability

Due to ethical restrictions, supporting data cannot be made openly available.
