# Supplementary material for: Impact of loneliness on health‐related factors in Australia during the COVID‐19 pandemic: A retrospective study
Source: Health Soc Care Community. 2022 Jul 28:10.1111/hsc.13948. Online ahead of print. doi: 10.1111/hsc.13948 (PMC9353389; doi:10.1111/hsc.13948)
Supplement: Supplementary file 1 — Appendix S1 [file HSC-9999-0-s001.docx]

**Supplementary Material**

**Instructions Provided to Respondents for the Online Survey Used for Data Collection**

We used identical set of questionnaires to collect participants before onset of COVID-19 pandemic and after the onset of COVID-19 pandemic data. Prior to every questionnaire, participants were presented with the following prompt in order to orient themselves and to maintain consistently and accuracy across responses. To capture before onset of COVID-19 pandemic data/responses, we used the prompt “*The next set of questions will ask you about your feelings and behaviours****BEFORE****the COVID-19 pandemic. Please focus on the time period before 11th of March 2020 to answer these questions*”. After the onset of COVID-19 pandemic questionnaires included the following prompts “*Please focus on your feelings****RIGHT NOW****”* or *“Please focus on your behaviours****CURRENTLY***”.

**Additional Details for Physical Health Measures**

Weight and Body Mass Index (BMI) - Participants self-reported their height and weight measurements including the metric used (for weight - kilograms or pounds and for height - centimetres or feet and inches). BMI values were calculated from the weight and height measurements using the formula BMI = Kilograms/metre ^2^.

Physical Activity - We asked participants to self-report their physical activity levels by asking them questions about the intensity (light, medium or high/vigorous) and the frequency of daily physical activity undertaken (e.g., *“How many days per week do you do* *light/medium/vigorous physical activities for at least 30 minutes?”).* We also provided definitions and examples of light, medium or high/vigorous intensity physical activity in the survey question for their edification. Scores for physical activity were calculated by creating dummy variables and assigning ‘values’ for intensity of physical activity (light = 1, medium = 2, vigorous = 3), and then multiplying the corresponding intensity ‘value’ with frequency of use per week (range 0 to 7). Scores ranged from 0 to 21, with higher scores indicating higher physical activity levels.

Alcohol Use – We asked participants to self-report their alcohol use by asking them whether they drank alcohol (intensity), possible responses included *-* never, rarely (less than once per week), regularly (at least once a week). Further, we asked them to report the number of standard drinks consumed per week (pictures depicting standard drink volumes for several alcohol beverages were included in the survey question). Scores for alcohol use were calculated by creating dummy variables and assigning ‘values’ for intensity of alcohol use (never = 0, rarely (less than once per week) = 1, regularly (at least once a week) = 2), and then multiplying the corresponding intensity ‘value’ with frequency of use (ranging from 1 standard drink per week to more than 10 (coded as 11)) per week. Scores ranged from 0 to 22, with higher scores indicating higher alcohol use.

**Data Cleaning, Screening, and Assumption Testing Information**

Eight hundred and two Australian residents (i.e., individuals residing in Australian at the time of the survey) completed the online survey during data collection. Of which 407 were community members and 395 were first year undergraduate students at Swinburne University. The data cleaning and analysis procedures were conducted using IBM Statistical Package for Social Sciences Version 27.0. Seven participants were excluded as they did not meet the inclusion criteria (i.e., they were under 18 years of age or not a current Australian resident). Further, 254 of the 802 participants had at least some incomplete survey responses (i.e., 32% of responses had at least some values missing). In order to obtain a complete data set, we employed multiple imputation (Markov Chain Monte Carlo method with 10 imputation models and 50 iterations using predictive mean matching; Eekhout et al., 2012; Heymans & Eekhout, 2019). Sensitivity analysis indicated missing data and multiple imputation did not affect the observed pattern of associations. Multiple imputation method yielded 700 complete set of responses. We further deleted 93 responses as they were either a univariate (z-score < ± 3.29) or a multivariate outlier (Mahalanobis distance ꭓ^2^ > 24.3; Field, 2013). The final data set included 607 participants, which was used in all the analyses included in the current paper.

The first research question - investigate the changes in physical health, social and mental health, as well as health literacy, of Australians before and during the COVID-19 pandemic was answered using paired samples *t-*tests. The second research question - associations between changes in loneliness and changes in health-related factors of Australians subsequent to the onset of COVID-19 pandemic was answered using Hierarchical Multiple Regression. All the assumptions for paired samples t-tests and Hierarchical Multiple Regression were satisfied. Normality was assessed by visual inspection of histograms and analysing absolute and z-scores values for skewness and kurtosis for all physical health, social and mental health, and health literacy variables. There were some deviations from normality, however, in line with literature, given the large sample size, and since we had small to moderate deviations from normality for most of our variables (Field, 2013; Kim, 2013; Tabachnick & Fidell, 2007), no transformations were applied. As mentioned above, multivariate outliers with Mahalanobis distance (ꭓ^2^) of more than 24.3 were deleted. No evidence of multicollinearity and non-independence of errors were found (tolerance value > 0.3 and VIF < 3; Durbin Watson statistic between 1.5 and 2.5 for all outcome/dependent variables; Field, 2013; Tabachnick & Fidell, 2007).

**References**

Eekhout, I., de Boer, R. M., Twisk, J. W., de Vet, H. C., & Heymans, M. W. (2012). Missing data: A systematic review of how they are reported and handled. Epidemiology, 23(5), 729-732. https://doi.org/10.1097/EDE.0b013e3182576cdb.

Field, A. (2013). *Discovering statistics using IBM SPSS statistics*. Sage.

Heymans, M. W., & Eekhout, I. (2019). *Applied Missing Data analysis with SPSS and R studio*. https://bookdown.org/mwheymans/bookmi/

Kim, H.Y. (2013). Statistical notes for clinical researchers: Assessing normal distribution (2) using skewness and kurtosis. *Restorative Dentistry & Endodontics*, *38*(1), 52-54. https://doi.org/10.5395/rde.2013.38.1.52

Tabachnick, B. G., & Fidell, L. S. (2007). *Using multivariate statistics* (5^th^ ed.). Allyn & Bacon/Pearson Education.
